# 

**Published:** 1887-06

**Authors:** 


					﻿OLD SERIES
VoL XXV
1855
' X E
NEW SERIS^I
Vol. IV.-No.




BkVJw
bjr^ w.f.westmor^^mdjH
H.V.M.M1LLER.M.D.LL.D.JF
’	JAME^A.GRAY.MJ}.1
MuBUSHED By JAMES pjharrison&c^
IK	<SaTLANTA.GA.
SUBSCRIFTIONs S2.BO YEAR.
				

